# Tau Protein in Oral Mucosa and Cognitive State: A Cross-sectional Study

**DOI:** 10.3389/fneur.2017.00554

**Published:** 2017-10-13

**Authors:** Luis Fernando Arredondo, Saray Aranda-Romo, Ildefonso Rodríguez-Leyva, Erika Chi-Ahumada, Sami K. Saikaly, Diana P. Portales-Pérez, Roberto González-Amaro, Mariana Salgado-Bustamante, Lourdes Enriquez-Macias, William Eng, Robert A. Norman, Maria E. Jimenez-Capdeville

**Affiliations:** ^1^Departamento de Bioquímica, Facultad de Medicina, Universidad Autónoma de San Luis Potosí, San Luis Potosí, Mexico; ^2^Clínica de Diagnóstico, Facultad de Estomatología, Universidad Autonóma de San Luis Potosi, San Luis Potosí, Mexico; ^3^Departamento de Neurología, Hospital “Ignacio Morones Prieto”, San Luis Potosí, Mexico; ^4^University of Central Florida College of Medicine, Orlando, FL, United States; ^5^Laboratorio de Inmunología Biologia Celular y Molecular, Facultad de Ciencias Químicas, Universidad Autónoma de San Luis Potosi, San Luis Potosi, Mexico; ^6^Centro de Investigación en Ciencias de la Salud y Biomedicina, Universidad Autónoma de San Luis Potosí, San Luis Potosí, Mexico

**Keywords:** tau protein, dementia, neurodegenerative diseases, Alzheimer disease, oral mucosa cells

## Abstract

Neurodegenerative diseases are characterized by the presence of abnormal aggregates of proteins in brain tissue. Among them, the presence of aggregates of phosphorylated Tau protein (p-Tau) is the hallmark of Alzheimer’s disease (AD) and other major neurodegenerative disorders such as corticobasal degeneration and frontotemporal dementia among others. Although Tau protein has previously been assumed to be exclusive to the central nervous system, it is also found in peripheral tissues. The purpose of this study was to determine whether there is a differential Tau expression in oral mucosa cells according to cognitive impairment. Eighty-one subjects were enrolled in the study and classified per Mini-Mental State Examination test score into control, mild cognitive impairment (MCI), and severe cognitive impairment (SCI) groups. Immunocytochemistry and immunofluorescence revealed the presence of Tau and four p-Tau forms in the cytoplasm and nucleus of oral mucosa cells. More positivity was present in subjects with cognitive impairment than in control subjects, both in the nucleus and cytoplasm, in a speckle pattern. The mRNA expression of Tau by quantitative real-time polymerase chain reaction was higher in SCI as compared with the control group (*P* < 0.01). A significantly higher percentage of immunopositive cells in the SCI group was found *via* flow cytometry in comparison to controls and the MCI group (*P* < 0.01). These findings demonstrate the higher presence of p-Tau and Tau transcript in the oral mucosa of cognitively impaired subjects when compared with healthy subjects. The feasibility of p-Tau quantification by flow cytometry supports the prospective analysis of oral mucosa as a support tool for screening of proteinopathies in cognitively impaired patients.

## Introduction

Dementia, or major neurocognitive disorder, is characterized by the deterioration of memory and intellect, alterations of behavior, and loss of the ability to perform everyday activities. Neurodegenerative diseases account for most of the cases of dementia (1) among them, Alzheimer’s disease (AD) represents 60% of all cases of dementia (2). The progressive brain damage that leads to dementia in these illnesses is associated with the presence of abnormal aggregates of proteins in brain tissue. Therefore, they are referred to as proteinopathies (3). The presence of neurofibrillary tangles formed by misfolded and aggregated Tau protein is the histopathologic hallmark of AD, frontotemporal lobar dementia, and corticobasal degeneration, among other tauopathies (4, 5).

While phosphorylation regulates Tau functions such as stabilizing microtubules and promoting their assembly and dynamic stability, the aggregation of Tau has been linked to an abnormal phosphorylation and the ability to autocatalyze the formation of new hyperphosphorylated tangles (6). Tau hyperphosphorylation has several deleterious effects in neurons, besides those related to cytoskeletal dysfunction caused by its detachment from microtubuli, such abnormal anterograde and retrograde movement of motor proteins and their cargos to and from the synapse. p-Tau undergoes re-localization to dendritic spines, where it compromises synaptic function (7). Also, altered protein synthesis has been mainly attributed to p-Tau interaction with ribosomes (8), to the triggering of the unfolded protein response pathway (9) and by its effect in the nucleus, where DNA fragmentation, loss of heterochromatin, activation of cell cycle, and alterations in the nuclear architecture (10) have been demonstrated. All these neurotoxic effects of p-Tau result in synaptic loss of vulnerable neurons from selected brain regions and may underlie the close association between Tau aggregation and cognitive deficit (11). Importantly, Tau aggregates can exit from one neuron to be internalized by other, which results in the spread of the proteinopathy across brain cells (12, 13). This phenomenon of the dissemination of an abnormal protein is the prion-like characteristic of all proteinopathies (14). The gradual synaptic loss progresses to cell death due to alterations in proteostasis caused by the building up and inclusion of Tau aggregates, which mainly affects protein clearance through the ubiquitin proteasome system and autophagy (15).

Although proteinopathies have previously been assumed to be exclusive to the central nervous system (CNS), several recent studies demonstrate their presence in peripheral tissue. Some examples include the skin and gut (16), for alpha-synuclein, in the case of Parkinson’s patients, p-Tau in the skin of AD patients (17, 18) and plasma, where several forms of p-Tau have been extracted from neurally derived blood exosomes (19). These findings strongly suggest that proteinopathies are systemic diseases whose fingerprints could be tracked in an accessible peripheral tissue. Currently, few studies have explored the presence of Tau in oral mucosa epithelium. Some cytological parameters, including cell type ratio and DNA content/neutral lipids ratio, were evaluated and associated with AD in the oral mucosa (20). Nevertheless, the fact that cytological parameters can be affected by exposure to genotoxic agents undermines this possible relationship to a neurodegenerative process (21). A previous study reported higher levels of p-Tau in AD patients as compared with healthy controls in oral mucosa epithelium and cerebrospinal fluid (CSF) by ELISA (22). More recently, the presence of another hallmark of AD, the beta-amyloid peptide (Aβ), assessed by laser scanning cytometry, was reported to be higher in the oral mucosa of AD patients than in healthy controls (23).

Considering that oral mucosa cells are accessible by exfoliative cytology through a non-invasive procedure and that they share a common embryonic origin with the skin and CNS, these advantages open the possibility to study proteinopathies in human peripheral tissue. Oral mucosa cells could allow elucidating whether the presence and localization of p-Tau is altered as compared to healthy subjects. In addition to providing means for the study of Tau dynamics, oral mucosa cells could also serve for adjunctive confirmation of a proteinopathy. The diagnosis of AD has evolved in parallel with the development of more sophisticated and useful imaging techniques (24). Initially, and still in use, diagnosis involved the clinical history of the patient supported by evidence of atrophy and structural changes measured through magnetic resonance imaging. Currently, positron emission tomography (PET) demonstrating cortical Aβ deposition is considered the more precise marker of AD associated neurodegeneration, although amyloidopathy is not exclusive of AD. The same situation applies to measures of hypometabolism by means of fluorodeoxyglucose tomography (25). More recently, the concentrations of Aβ42, total, and p-Tau in CSF have been found significantly associated with the presence of cognitive deficit; they are promising but still controversial and with limited clinical value (26–28). Since it is widely demonstrated that Tau pathology is more significantly associated with cognitive deficit than amyloidopathy, the development of the PET tracer flortaucipir, targeting tau aggregates, represents a promising tool for the early detection of tauopathies (29). Both image and immunoassay procedures confirm AD diagnosis, but their high cost and invasive nature imply an obstacle to population studies and to public health systems.

In light of these antecedents, the purpose of this study was to determine whether there is a differential Tau expression in oral mucosa cells according to cognitive impairment, as well as the development of a novel method based on flow cytometry to compare p-Tau presence among healthy and cognitively impaired individuals.

## Materials and Methods

### Subjects and Samples

This was a multicentric analytic cross-sectional study, carried out at the diagnostic clinic of the Universidad Autónoma de San Luis Potosí School of Dentistry, local private practice clinics in Mexico, and assisted living facilities in Tampa, FL, USA. The protocol was reviewed and approved by two Ethics committees (protocols CEI-FE-058-015 in Mexico and IRB solutions #2017/01/02 in Tampa, FL, USA). The subjects were enrolled consecutively and for convenience between January 2016 and January 2017, and they were always sampled by the same person. A total of 81 subjects older than 65 years, males and females, were included. Among them, neurocognitively impaired subjects included patients with probable AD diagnosed according to the NINCDS-ARDA criteria (30), patients with vascular dementia, and other neurodegenerative diseases. The informed consent was signed by themselves if possible; otherwise, the primary care provider or a relative signed on behalf of the subject. The participants accepted the application of the Mini-Mental State Examination test (MMSE) (31) and the donation of exfoliative cytology of oral mucosa. After explaining the purpose of the study and obtaining demographic information, two exfoliative samples were taken. Buccal cells were collected from the inner cheek by rotating the head of one of the cytological brushes 15 times in a circular motion. One cytological brush head was clipped into a tube containing 1 ml of ice-cold PBS, while the other brush was used to make a thin smear on a silane-coated microscope slide for fluorescence staining. The remaining sample on the brush, which was employed for light and fluorescence microscopy was also clipped into 1 ml of ice-cold PBS for nucleic acid extraction, in the 51 cases that were analyzed in Mexico. After the cells were detached from the cytological brush by vortex agitation for 15 s, the brush was removed and the sample centrifuged 5,000 × *g* for 5 min.

### Immunofluorescence

The presence and localization of tau protein in oral mucosa cells was analyzed by confocal microscopy. Two forms of phosphorylated Tau were examined, one epitope associated with normal and pathological Tau phosphorylation (17, 32) monoclonal rabbit anti p-Tau (Ser396) (Abcam, Cambridge, MA, USA, dilution 1:100) and a second epitope that characterizes Tau hyperphosphorylation monoclonal mouse anti p-Tau (Ser202 + Thr205, Thermo Fisher Scientific, Rockford, IL, USA dilution 1:100). Nuclear localization was explored employing monoclonal rabbit anti-Lamin-A (Abcam, Cambridge, MA, USA, dilution 1:100). Antibodies were incubated overnight at 4°C and then rinsed. For detection, goat α-rabbit Cy5 (Life Technologies, USA, dilution 1:200) and goat α-mouse Alexa Fluor 488 (Thermo Fisher Scientific, Rockford, IL, USA, dilution 1:300) were incubated for 2 h at room temperature, followed by SYTOX (Molecular Probes, Eugene, OR, USA, dilution 1:1,000) for 30 s, then rinsed and mounted with Vectashield^®^ (Vector Laboratories, Burlingame, CA, USA). Analysis was performed with a LEICA TCS SP2 confocal microscope (Leica Microsystems GmbH, Wetzlar, Germany).

### Immunocytochemistry

The slides were fixed in 4% paraformaldehyde for 10 min and washed with TBST (Tween^®^20 Tris-Buffered Saline). The slides were subjected to the following 15 min incubation steps alternating with TBST rinses, 3% hydrogen peroxide, Background Sniper (Biocare Medical, Pacheco, CA, USA) and avidin/biotin (Vector Laboratories, Burlingame, CA, USA). One primary monoclonal mouse antibody, anti p-Tau (Ser202 + Thr205, Thermo Fisher Scientific, Rockford, IL, USA dilution 1:20) and four primary rabbit monoclonal antibodies were assayed in oral mucosa cells, anti p-Tau (Ser202), anti p-Tau (Thr231), anti p-Tau (Ser214) (Abcam, Cambridge, MA, USA, dilution 1:80, 1:20, 1:20, respectively) recognize epitopes, which are present mostly in hyperphosphorylated Tau (33). The monoclonal mouse antibody Tau 5 (Thermo Fisher Scientific, Rockford, IL, USA, dilution 1:20), which stains non-phosphorylated region comprised between amino acids 210 and 241, was employed to examine total Tau. After 1 h incubation, a universal biotinylated link antibody (DAKO, Carpinteria, CA, USA) was incubated for 30 min, followed by streptavidin-HRP complex (DAKO, Carpinteria, CA, USA) for 30 min. The cytology was developed with 3-amino-ethyl-carbazole for 15 min, rinsed in water, counterstained with Mayer’s hematoxylin, and analyzed immediately. At least 25 fields per slide were captured with a NikonLabphot-2 microscope (Nikon Instruments, Melville, NY, USA) equipped with a digital camera in order to acquire 100–125 cells per sample. The digital analysis, based in the RGB (red, green, blue) model to detect positive cells, was performed with the Image Pro Plus 7 software (MediaCybernetics, Bethesda, MD, USA). The detected positive cells were manually counted, as the images were too complex for the software to discriminate single cells between clumps and mucus.

### Quantitative Real-time Polymerase Chain Reaction

Messenger RNA extraction was performed with TRIzol reagent (Thermo Fisher Scientific, Rockford, IL, USA) according to the manufacture’s protocol. All the samples were evaluated by spectrophotometry to assess the 260/280 ratio (>1.8). The synthesis of cDNA was performed with ImProm-II Reverse Transcription System (Promega Corp., Madison, WI, USA). Real-time PCR was performed with iQ SYBR Green Supermix (Bio-Rad Laboratories, Hercules, CA, USA) and 18S ribosomal subunit was used as housekeeping normalization gene (Fw/Rv, CGGCTACCACATCCAAGG/GCTGGAATTACCGCGGCT). The amplification of all six Tau isoforms, commonly referred as 4R-Tau and 3R-Tau due to the number of repetitions of the microtubule binding domain (4 and 3, respectively) were assessed with the following primer’s sequence: CGGGAAGGTGCAGATAATTAA/TATTTGCACACTGCCGCCT (Fw/Rv) for 4R-Tau and GGCGGGAAGGTGCAAATA/GCCACCTCCTGGTTTATGATG (Fw/Rv) for 3R-Tau. The data were computed by 2^−ΔCt^ method, where 2^−ΔCt^ = 2^−(Cq housekeeping − Cq Tau)^ (34).

### Flow Cytometry

Immediately after cell isolation, oral cells were transferred to a 5 ml cytometry tube and fixed (Fixation buffer, eBiosciences, Santa Clara, CA, USA) for 1 h at 4°C, then rinsed with buffer (eBiosciences, Santa Clara, CA, USA), and centrifuged 650 × *g* for 5 min. The supernatant was discarded and blocking was performed with 10% goat serum for 30 min at 4°C. After incubation with the primary antibody p-Tau (Ser202) for 1.5 h at 4°C, the cells were rinsed with permeabilization buffer and centrifuged 650 × *g* for 5 min. The pellet was incubated with the secondary antibody goat α-rabbit + FITC (EMD Millipore Corp., Temecula, CA, USA, dilution 1:10) for 30 min, rinsed with permeabilization buffer, and centrifuged 650 × *g* for 5 min. If the samples were programmed to be acquired later, 200 µl of 1% paraformaldehyde in PBS were added as a fixative. A no-stain sample and the secondary antibody-only sample were prepared to assess the unspecific binding of the secondary antibody.

### Statistical Analysis

Demographic and clinical characteristics were analyzed using descriptive statistics. All data are presented as mean ± SD. The participants were divided in three groups according to their MMSE score as follows: control group (CTRL) 24–30 score points, mild cognitive impairment (MCI) 18–23 score points, and severe cognitive impairment (SCI) 0–17 score points. Data were analyzed using one-way ANOVA with Tukey’s multiple comparison posttest, except for the 4R-Tau relative expression, which was analyzed using Kruskal–Wallis test with Dunn’s multiple comparison posttest. Differences between categorical variables were evaluated using a Fisher’s exact test. The dependence analysis was performed using Spearman’s rank correlation coefficient. A *P*-value <0.05 was considered statistically significant. All statistical analyses were performed with GraphPad Prism 6.0 (GraphPad Software Inc., San Diego, CA, USA).

## Results

A total of 81 subjects were enrolled in the study, 71% were from central Mexico, where the population is the result of a mix of Hispanic and indigenous native (Mexican mestizo) populations, while 29% were living in Tampa, FL, USA, most of whom were Caucasian participants. Baseline characteristics according to their MMSE score are shown in Table 1. There is a higher frequency of women in the SCI groups as compared to MCI and control groups. This difference could be attributable to the higher life expectancy and incidence of some types of dementias among the female population. Sixteen patients with clinical diagnosis of AD were included in the two groups of cognitively impaired subjects, as AD is the most common and prototypical tauopathy.

**Table 1:** Demographic characteristics of the population according to Mini-Mental State Examination test (MMSE) score.

Variable	Groups			
	C (*N* = 31)	MCI (*N* = 16)	SCI (*N* = 34)	Total (*N* = 81)
	
	*N* (%)	*N* (%)	*N* (%)	*N* (%)
**Sex**				
Female	13 (23.6)	13 (23.6)	29 (52.7)	55 (100)*
Male	18 (69.2)	3 (11.5)	5 (19.2)	26 (100)*
Age (mean ± SD)	74.5 ± 7.5	82 ± 6.1	84.1 ± 7.5	80.1 ± 8.4^
MMSE (mean ± SD)	28.1 ± 2.2	19.5 ± 1.9	10.5 ± 5.1	19 ± 8.7^

*C, control; MCI, mild cognitive impairment; SCI, severe cognitive impairment; MMSE, Minimental Status Evaluation*.

*P < 0.0001 Fisher exact test for categorical variables.

*^P < 0.001 ANOVA followed by Tukey’s multiple comparisons test for the variables age and MMSE*.

### Tau Protein Localization in Oral Mucosa Cells

**Figure 1:**
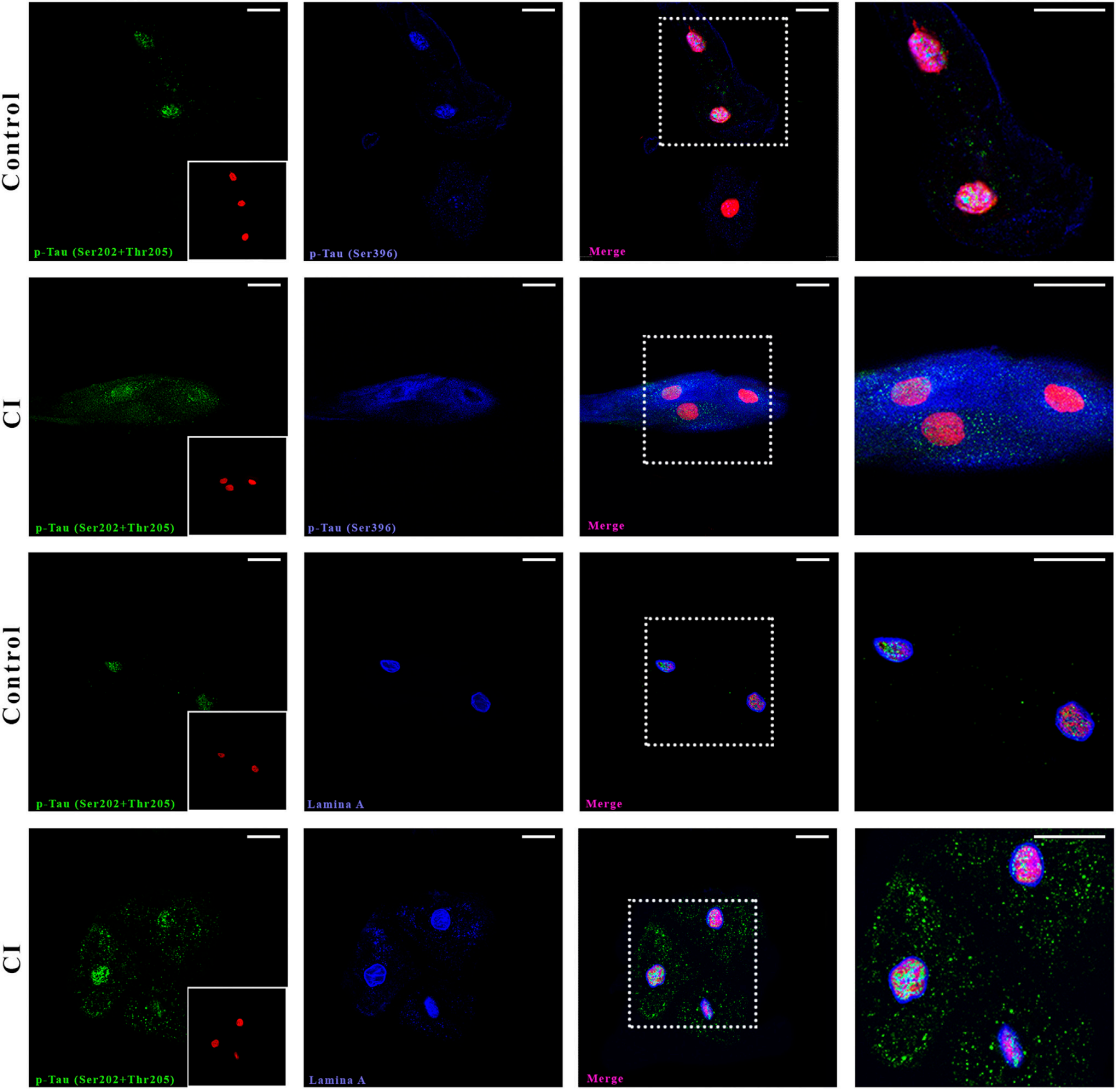
Confocal microscopy. Presence and distribution of p-Tau in oral mucosa cells. Control and cognitive impaired (CI) subject. The two first rows show p-Tau (Ser202 + Thr205) and p-Tau (Ser396) immunostaining. The following two rows depict the juxtanuclear localization of p-Tau (Ser202 + Thr205) in nucleus (Lamin A, blue). The nuclei stained with SYTOX (red) are shown in a box in the first image of each series. The scale bars represent 20 µm for all images.

First, we searched by confocal microscopy whether two phosphorylated forms of Tau were present in mucosa cells of one control and one SCI patient diagnosed with AD (Figure 1). Both phosphorylated forms were present in the cytoplasm and nucleus of oral mucosa cells. Immunopositivity of the most common p-Tau found in pathological conditions [p-Tau (Ser202 + Thr205)] was more intense in the patient than in the control subject (Figure 1, first and second row, green), especially in nuclei. The phosphorylated form p-Tau (Ser396) was localized mainly in the cytoplasm and around the nucleus and showed also more positivity in the patient than in the control subject. The nuclear localization of p-Tau was confirmed by utilizing a marker of the nucleoskeleton, Lamin A (Figure 1, third and fourth row, blue), in addition to the nuclear marker SYTOX, which binds to nucleic acids (Figure 1, red marker). Both nuclear markers had similar distribution and immunopositivity in the SCI patient and the control subject, while p-Tau clearly shows a higher intensity in the SCI patient, both in nucleus and cytoplasm in a speckle pattern.

**Figure 2:**
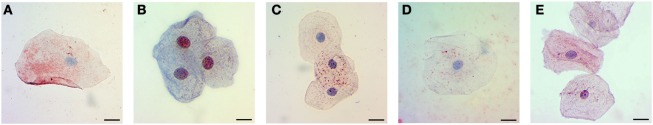
Immunocytochemistry for Tau and p-Tau in oral mucosa cells. Assay of different monoclonal antibodies. **(A)** Tau-5. **(B)** p-Tau (Ser396). **(C)** p-Tau (Thr231). **(D)** p-Tau (Ser214). **(E)** p-Tau (Ser202 + Thr205). The scale bars represent 20 µm for all images.

**Figure 3:**
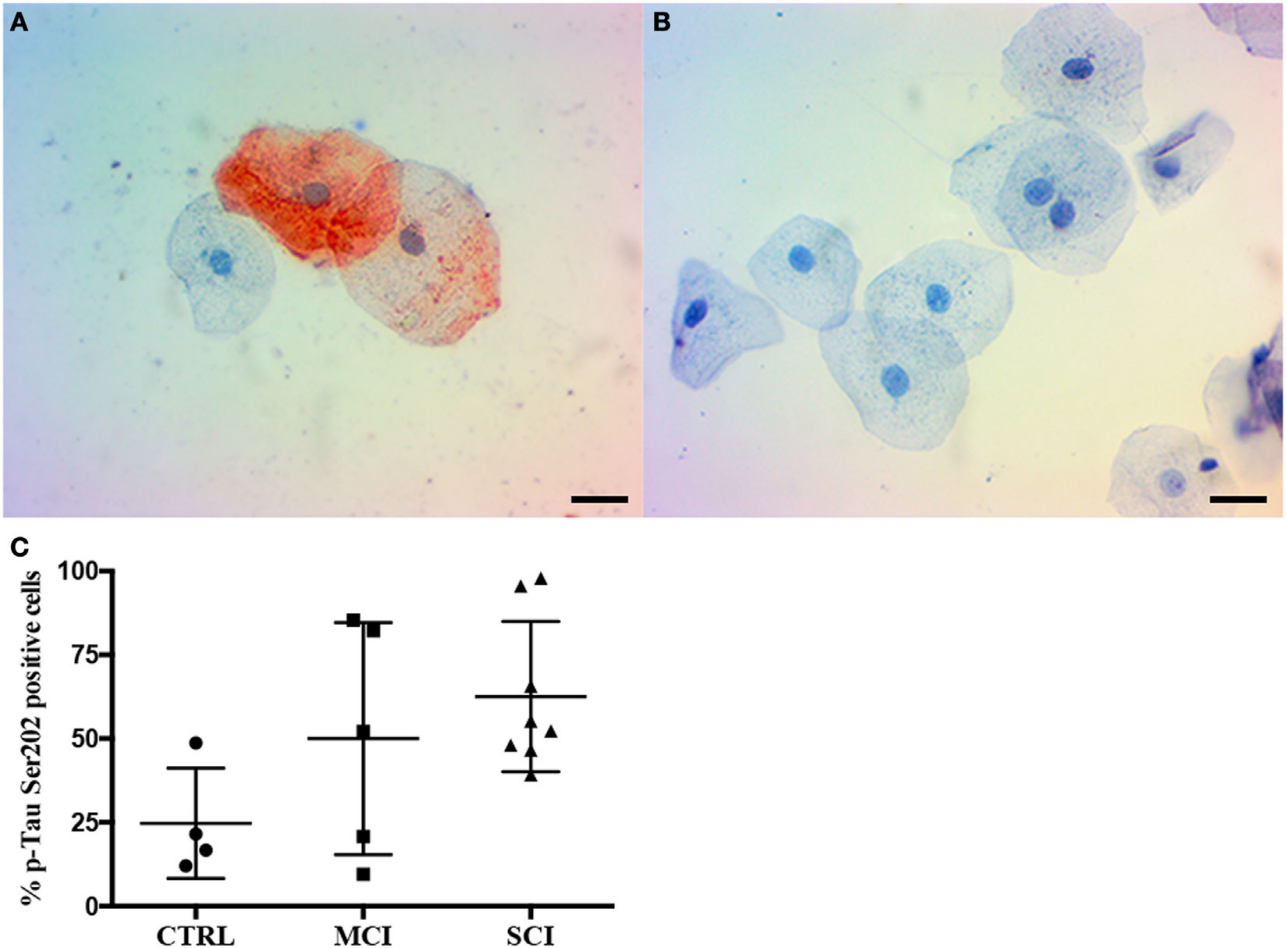
Immunocytochemistry for p-Tau in oral mucosa cells. **(A,B)** Immunocytochemistry staining shows p-Tau (Ser202) presence in oral mucosa, **(A)** cognitive impaired subject, **(B)** control subject. **(C)** p-Tau (Ser202) positivity percentage by cognitive state. The scale bars represent 20 µm for all images. Mean plus SD, *P* = 0.108.

The phosphorylation site employed to compare Tau presence in oral mucosa cells from control subjects and patients with dementia was selected after assaying four anti-p-Tau monoclonal antibodies. Figure 2 shows that the phosphorylation independent antibody Tau 5 reveals immunopositivity mostly in cytoplasm, while anti p-Tau antibodies, p-Tau (Ser396), p-Tau (Thr231), and p-Tau (Ser214) stained both the cytoplasm and the nucleus in a cognitive impaired subject. The best signal was obtained with anti Tau (Ser202), both for immunocytochemistry (Figure 3) and flow cytometry.

Given these results, the second phase of the study focused on the evaluation of immunopositivity to p-Tau (Ser202) by immunocytochemistry. Examples from a control subject and a SCI patient are shown in Figure 3. A total of 17 samples were included and distributed as follows: control subjects (4), MCI (5), and SCI (8). The immunocytochemistry shows p-Tau presence both in the cytoplasm and nucleus of oral mucosa cells, and a higher immunopositivity in the dementia cases. The result is displayed in Figure 3C. While a tendency toward higher immunopositivity in MCI (50 ± 34.6%) and SCI (62.5 ± 22.4%) groups is present when compared to controls (24.7 ± 16.4%), it did not reach statistical significance (*P* = 0.108).

### Cognitively Impaired Subjects Present Increased Tau Transcriptional Activity and Higher Levels of p-Tau in Oral Mucosa Cells

**Figure 4:**
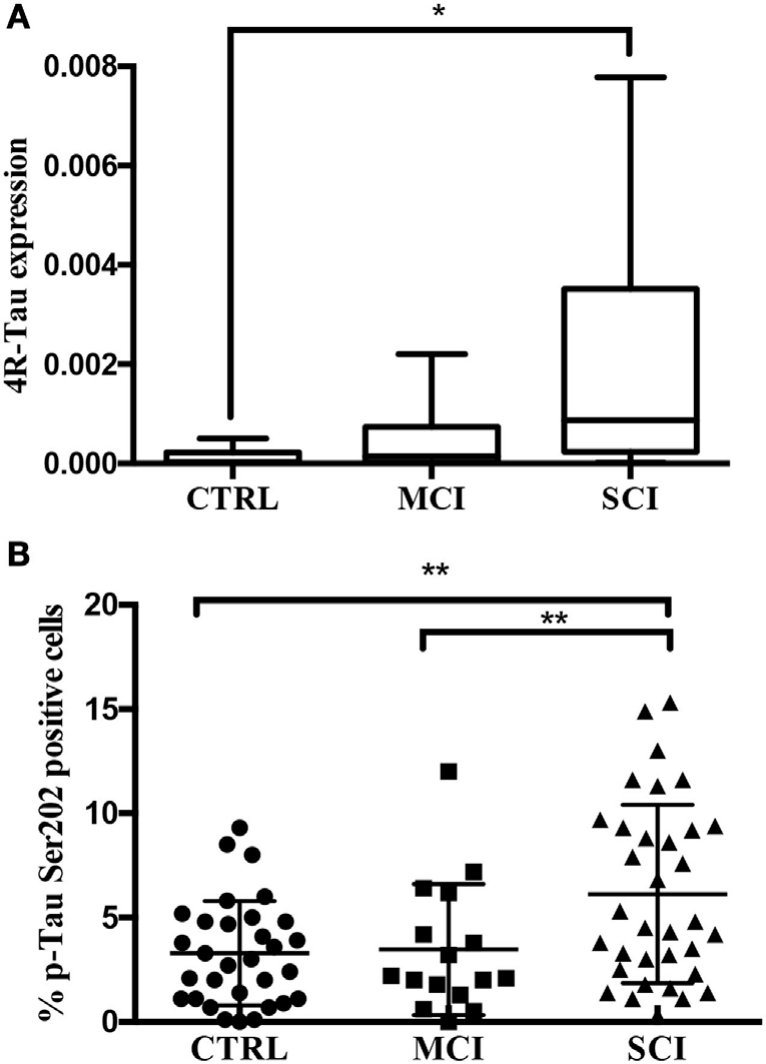
**(A)** 4R-Tau transcript evaluated by quantitative real-time polymerase chain reaction. 4R-Tau relative expression levels (2^−ΔCq^) and normalized to 18s ribosomal subunit. Line: median, whiskers: min–max, box: interquartile range. *P* < 0.01. **(B)** p-Tau positivity by flow cytometry. Percentage of p-Tau (Ser202) positive oral mucosa cells by cognitive state. Mean plus SD, **P = 0.0029.

Once the presence of the protein was detected in oral mucosa cells, the next step was to investigate whether mRNA expression level is altered in cognitively impaired states, since the phosphorylation of Tau is a posttranslational modification. A relative expression analysis was performed in 51 subjects using the 18s ribosomal subunit as a housekeeping gene and two set of primers allowing the amplification of all six Tau isoforms, 4R-Tau and 3R-Tau. This analysis was performed only in the 51 participants from Mexico, because the lab facilities in Tampa, FL, USA did not include equipment for nucleic acid analysis. The data are expressed in arbitrary units as median (min–max), CTRL: 3.2 × 10^−5^ (3.1 × 10^−6^ to 0.5 × 10^−4^). MCI: 1.4 × 10^−4^ (3.8 × 10^−6^ to 2.2 × 10^−3^). SCI: 8.6 × 10^−4^ (1 × 10^−5^ to 7.8 × 10^−3^). As shown in Figure 4A, a statistical difference was present between the control and SCI group (*P* < 0.01) and a slight increase in relative expression in MCI group. The isoforms 3R-Tau were not expressed in oral mucosa cells (Figure S1 in Supplementary Material).

Next, in order to overcome the difficulties regarding the quantification of p-Tau-positive cells by immunohistochemistry, this study presents a novel approach based on flow cytometry. In contrast with the semiquantitative results obtained by computerized image analysis of immunopositivity, flow cytometry provides quantitative data. The suspension of single cells was achieved by vigorous vortex agitation and confirmed by light microscopy. A significantly higher percentage of p-Tau positive cells was present in SCI (6.1 ± 4.2%) than in MCI (3.4 ± 3.1%) and control group (3.2 ± 2.5 5) (*P* < 0.01; Figure 4B). The main advantage of this quantification method is the number of analyzed cells (10,000 per sample). This provides a more reliable landscape than the histocytochemical/digital processing analysis, which can be susceptible to bias due to the selection of photographed fields and background. Finally, no correlation was found between the percentage of p-Tau positive cells and age (Rho = 0.096, *P* = 0.396; Figure S2 in Supplementary Material).

## Discussion

With the analysis of Tau protein expression in oral mucosa cells obtained from healthy subjects, MCI and SCI patients, this study demonstrates the differential expression of Tau mRNA and phosphorylated protein in dementia by means of immunocytochemistry, flow cytometry, and qPCR. The developed method of Tau quantification in oral mucosa cells through flow cytometry offers a new approach to neurodegeneration research. By a simple brushing of the oral mucosa, an appropriate sample of epithelial tissue is obtained, and these cells have the same ectodermal origin of the nervous system cells (35). The main short-term application of this method arises from the demonstration of a higher percentage of p-Tau positive cells in individuals with a low MMSE score. Therefore, a patient with cognitive impairment and a high percentage of p-Tau positive cells is certainly suffering a proteinopathy and very possibly a neurodegenerative disease. Moreover, the expression of Tau messenger RNA can be quantified by qPCR in the same sample and a high value of this parameter will confirm the presence of a proteinopathy.

It is a relevant finding that p-Tau does not form tangles in oral mucosa cells. The analysis of p-Tau through confocal microscopy revealed a punctuated pattern of staining, similar to that observed in HEK-293 cells exposed to p-Tau fibrils obtained from brain tissue of deceased AD patients (14). In contrast, when these fibrils were injected into mice hippocampus the classic tangle patterns appeared. Since similar punctuated patterns of p-Tau immunostaining have been reported in lymphocytes (36), fibroblasts (37), and skin cells (18), this indicates a distinctive feature of p-Tau in no-neural tissue, and it may be related to different functions of Tau in peripheral tissue. Also, immunocytochemistry confirmed the same pattern and localization of p-Tau (Figures 2 and 3).

It is also of interest that our findings employing immunohistochemistry and flow cytometry in a population of Caucasians and Mexican mestizo subjects are in agreement with the data found by Hattori et al. (22) in Japanese subjects. Using ELISA determinations in oral mucosa cells, they reported higher concentration of Tau in AD patients than in age-matched healthy controls, both in oral mucosa cells and CSF. Also, similar to our study was the fact that they did not find a significant correlation between Tau concentration in oral mucosa cells and age. On the other hand, also by means of ELISA, François et al. (23) did not find a significant difference between controls, MCI subjects, and AD patients in an Australian population. They may have had a different outcome from a study similar to ours because we searched p-Tau instead of total Tau, and our SCI sample included patients with several neurodegenerative diseases besides AD, whose high p-Tau values were accompanied by very low MMSE scores.

Currently, it is widely demonstrated that Tau dysfunction in the CNS causes neurodegeneration, although the multiple roles of this protein in healthy conditions have not been fully elucidated. Activation of numerous signaling pathways leads to Tau phosphorylation, and hyperphosphorylation in diseased states reflects an increase in the proportion of Tau molecules that are phosphorylated at given amino acids. The antibodies used in this work detect phosphorylated sites that constitute a signature of Tau hyperphosphorylation. In brain tissue, phosphorylation of the epitope Ser202-Thr205 is useful to detect neuritic plaques, a clear sign of neurodegeneration (Figure S3 in Supplementary Material, left), and anti p-Tau (Ser396) shows more positivity in axons and dendrites than the antibody against total Tau (Figure S3 in Supplementary Material, center and right, respectively). However, the meaning of the observed pattern of Tau phosphorylation in oral mucosa cells is uncertain, mainly because the physiological role of Tau in these cells is not known yet. Experimental evidence of its interaction with cytoskeleton is still lacking, but since in neurons Tau phosphorylation plays a key role in plasticity and axonal transport, and these features are absent in oral mucosa cells, certainly Tau plays other functions among them. Some topics of discussion that arise from these results are where and how increased Tau phosphorylation starts in these cells and whether p-Tau propagates between them (13, 38). The understanding of p-Tau spreading is still in progress, and a clear example is that some regions in human brain predicted to be free of p-Tau deposition have the possibility to induce Tau aggregation *in vitro* (6). Unfortunately, it is not possible to determine whether the presence of p-Tau in oral mucosa has pathologic significance. Nevertheless, we cannot neglect the possible role of oral mucosa in p-Tau spreading.

In addition to representing a very useful model for the analysis of p-Tau localization, flow cytometry quantification of p-Tau can be employed to support the diagnosis of a proteinopathy, although not with the purpose of a differential diagnosis from other tauopathies, until a more detailed profile of p-Tau in different neurodegenerative diseases will be defined.

In conclusion, these findings demonstrate the higher presence of p-Tau and 4R-Tau transcript in the oral mucosa of cognitively impaired patients than in that of healthy subjects. Additionally, the feasibility of p-Tau quantification by flow cytometry supports the prospective analysis of oral mucosa as a support tool for screening of proteinopathies among cognitively impaired patients.

## Ethics Statement

This study was carried out in accordance with the recommendations of Ley General de Salud, México, 2011, CONBIOETICA with the written informed consent from all subjects. All subjects gave written informed consent in accordance with the Declaration of Helsinki. The protocol was approved by two Ethics committees, CEI-FE-058-015 in Mexico and IRB solutions #2017/01/02 in Tampa, FL, USA.

## Author Contributions

LA, SA-R, IR-L, and MJ-C contributed to conception, design, data acquisition, analysis and interpretation, drafted and critically revised the manuscript. EC-A, MS-B, LE-M, DP-P, RG-A, WE, and RN contributed to data acquisition, analysis, and critically revised the manuscript. SS contributed to data acquisition, drafted and critically revised the manuscript. All authors gave final approval and agreed to be accountable for all aspects of the work.

## Conflict of Interest Statement

The authors declare that the research was conducted in the absence of any commercial or financial relationships that could be construed as a potential conflict of interest.
