# Supplementary material for: Tau Protein in Oral Mucosa and Cognitive State: A Cross-sectional Study
Source: Front Neurol. 2017 Oct 13;8:554. doi: 10.3389/fneur.2017.00554 (PMC5645496; doi:10.3389/fneur.2017.00554)
Supplement: Supplementary file 1 [file Data_Sheet_1.DOCX]

Supplementary Material

Tau protein in oral mucosa and cognitive state:

A cross-sectional study

# Luis F. Arredondo MSc^1^, Saray Aranda-Romo PhD^2^, Ildefonso Rodríguez-Leyva MD, PhD, FAAN^3^, Erika Chi-Ahumada BSc^1^, Sami K. Saikaly MD^4^, Diana P. Portales-Pérez PhD^5,7^, Roberto González-Amaro MD, PhD^6^, Mariana Salgado-Bustamante PhD^1^, Lourdes Enriquez-Macias MSc^1^, William Eng MD^4^, Robert A. Norman D.O^4^, Maria E. Jimenez-Capdeville PhD^1*^.

* Corresponding author at: Facultad de Medicina, Universidad Autónoma de San Luis Potosí. Av. V. Carranza 2405 Col. Los Filtros, 78210 San Luis Potosí S.L.P., México. Phone: +52 (444) 826 23 00 Ext. 6630 e-mail: [mejimenez@uaslp.mx](mailto:mejimenez@uaslp.mx)

## Supplementary Figures

**Supplementary Figure 1.** **Tau 3R and 4R mRNA expression**. **(Left panel)** 3R-Tau amplification plot. We found 0% of positive samples for 3R-Tau mRNA, the representative plot includes 2 samples from cultured fibroblasts as positive control. (**Center panel)** 4R-Tau amplification plot. The representative plot includes 2 samples from cultured fibroblasts as positive control. (**Right panel)** Total percentage of amplifying samples for 18s (housekeeping gene), 4R-Tau and 3R-Tau. All the q-PCR experiments were carried by duplicate, this representative experiment includes 5 samples per group (blue), including no template control (light blue) and positive controls (red).

**Supplementary Fig 2.** **Relationship between p-Tau immunopositivity and age.** The control group and the cognitive deficit groups were divided in two age ranges, 69-79 and > 80 and p-Tau immunopositivity was evaluated according to the group (**left and center panels**). All p-Tau immunopositivity data were plotted versus age and analyzed by Spearman correlation (**right**). No significant association between age an p-Tau immunopositivity was found.

**Supplementary Fig. 3. Patterns of Tau immunostaining in human brain tissue.** Mouse monoclonal anti Tau (Tau5, left). Rabbit monoclonal anti p-Tau(Ser396) (center), and mouse monoclonal anti p-Tau (Ser202+Thr205) (right) were assayed in paraffin included tissue sections (5uM) of autopsied human brain. Neurofibrillary tangles are observed in all panels. Arrows point to neuritic plaques. Scale bars: 10uM
