# Supplementary figures and images for: Tau Protein in Oral Mucosa and Cognitive State: A Cross-sectional Study
Source: Front Neurol. 2017 Oct 13;8:554. doi: 10.3389/fneur.2017.00554 (PMC5645496; doi:10.3389/fneur.2017.00554)

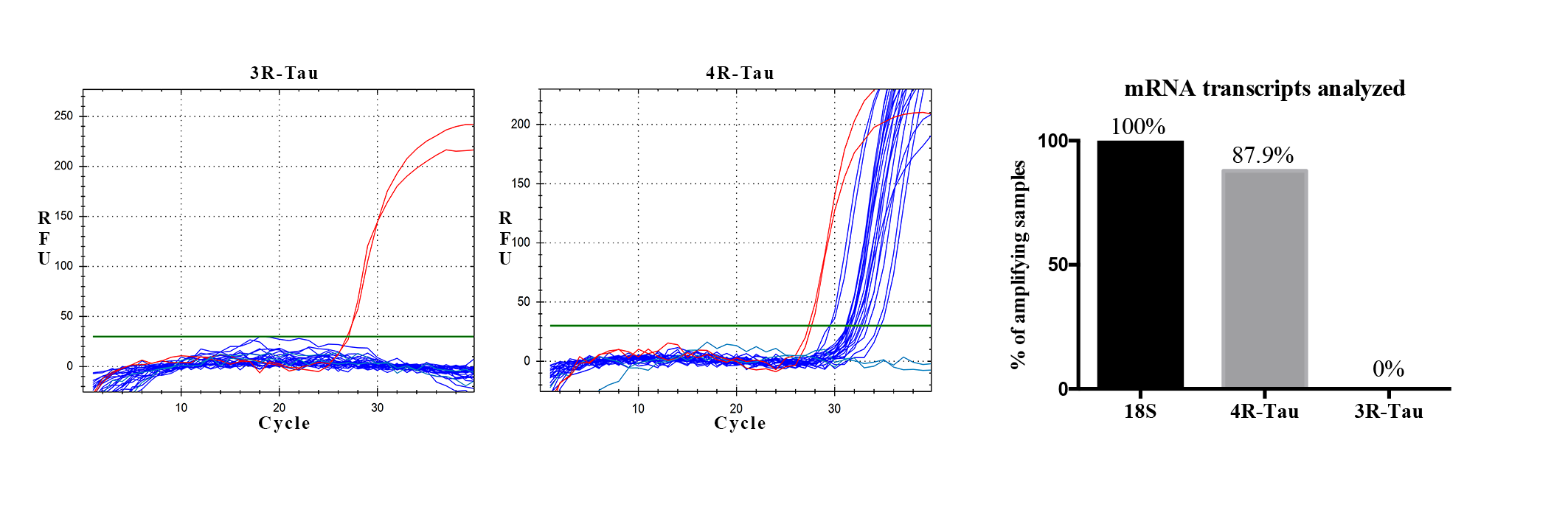

Supplement: Supplementary file 2 [file Image_1.TIF]

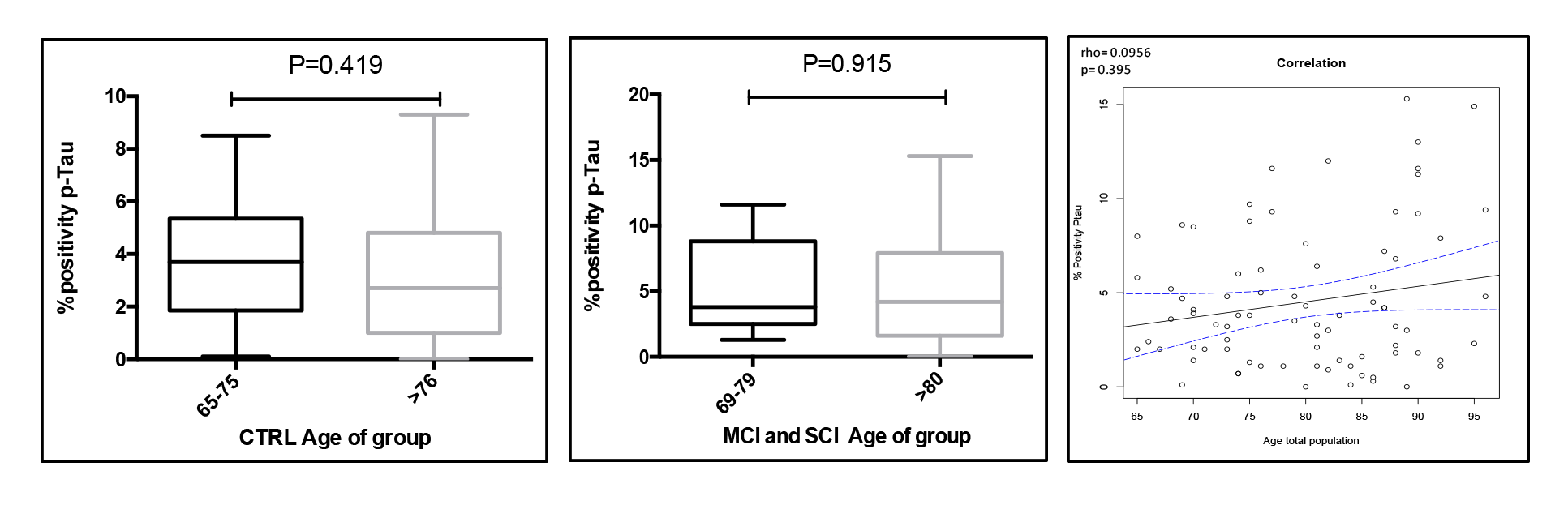

Supplement: Supplementary file 3 [file Image_2.TIF]

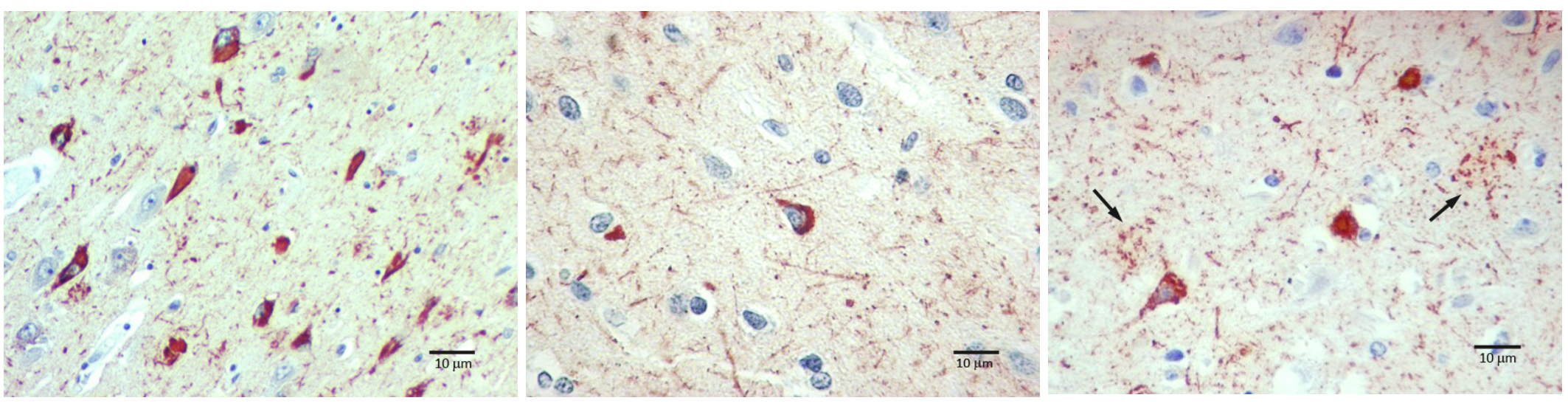

Supplement: Supplementary file 4 [file Image_3.TIF]
